# Gene Signatures and Prognostic Values of m^6^A Genes in Nasopharyngeal Carcinoma

**DOI:** 10.3389/fonc.2020.00875

**Published:** 2020-06-11

**Authors:** Shanshan Lu, Zhengzheng Yu, Zhiqiang Xiao, Yiya Zhang

**Affiliations:** ^1^Research Center of Carcinogenesis and Targeted Therapy, Xiangya Hospital, Central South University, Changsha, China; ^2^The Higher Educational Key Laboratory for Cancer Proteomics and Translational Medicine of Hunan Province, Xiangya Hospital, Central South University, Changsha, China; ^3^National Clinical Research Center for Geriatric Disorders, Xiangya Hospital, Central South University, Changsha, China; ^4^Department of Dermatology, Xiangya Hospital, Central South University, Changsha, China

**Keywords:** nasopharyngeal carcinoma, prognostic factors, m^6^A, METTL3, immune infiltration

## Abstract

Nasopharyngeal carcinoma (NPC) is a malignant tumor with a high rate of local invasion and early distant metastasis. Accumulating studies suggest that N6-methyladenosine methylation (m^6^A) is closely related to tumorigenesis. However, the relationship between m^6^A-related genes and prognosis of NPC is poorly understood. Our research aims to discover the prognostic value of m^6^A RNA methylation genes in NPC. In this study, we analyzed the differentially expressed m^6^A-related genes between NPC samples and normal control samples and found that two upregulated genes (YTHDF3 and IGF2BP2) and one downregulated gene (METTL3) were overlapped in GSE68799 and GSE53819. Next, we found that high expression of IGF2BP1 and low expression of METTL3 and YTHDF3 in NPC patients showed poor progression-free survival (PFS). Subsequently, the four m^6^A genes were selected for consensus cluster analysis, and risk models were established. The risk signature, using three genes (GF2BP1 + IGF2BP2 + METTL3), was an independent prognostic factor and predicts the clinicopathological features of NPC. Additionally, the GO, KEGG analysis, and CIBERSORT algorithm revealed that the risk signature was closely associated to immune infiltration in NPC. Finally, the expression and clinical significance of METTL3 were successfully validated in NPC tissues using immunohistochemical techniques. In conclusion, our finding revealed the potential role of m^6^A modification in NPC, providing novel insight into NPC prognosis and therapeutic strategies.

## Introduction

Nasopharyngeal carcinoma (NPC), which occurs in squamous epithelial cells of the nasopharyngeal mucosa (1), has a particularly high prevalence (2) in central and southern coastal areas of China and Southeast Asian countries (3, 4). Early symptoms of NPC are difficult to identify (5, 6). Most patients with NPC are in the middle and advanced stages at the time of diagnosis, and even lymph nodes and distant organ metastases have occurred (7, 8), with the 5-year survival rate <20% (9, 10). Therefore, it is urgent to identify reliable prognostic molecular markers and accurately predict the prognosis of NPC.

N6-methyladenosine methylation (m^6^A) is a post-transcriptional modification of RNA that occurs on the N atom at position 6 of RNA adenine (A) (11). To date, more than 170 post-transcriptional modifications of RNA have been discovered (12), and m^6^A is the most common and plentiful modification (13) and is ubiquitous in eukaryotes (14). The m^6^A modification is deposited by “writer” (methyltransferase), demethylated by “erase” (demethylase), and executed by “reader” (binding protein or recognition protein) process (12, 15). Methyltransferase catalyzes m^6^A methylation of RNA, and its core members are METTL3, METTL14, WTAP, etc. (16). Demethylase mediates m^6^A demethylation modification, with core members FTO and ALKBH5 (17). Binding proteins recognize RNA methylation modifications, including the YTHDF family (YTHDF1/2/3), nuclear heterogeneous riboprotein family (HNRNPA2B1, HNRNPC), and eukaryotic initiation factor (eIF) (15, 16). m^6^A methylation is involved in RNA metabolism-related processes such as RNA transcription, processing, splicing, degradation, and translation (18, 19). A recent study revealed that the dysregulated m^6^A-related genes are involved in the progression of various cancers (20). METTL3/METTL14 play a vital role in the progression of glioblastoma (21), breast (22), and liver cancer (23); FTO, notorious fat mass and obesity-associated protein, is related to the occurrence of leukemia (24), thyroid cancer (25), breast cancer, and gastric cancer (26); YTHDF2 promotes the proliferation and migration of pancreatic (27) and prostate cancer cells (25).

However, the effect of m^6^A methylation modification on NPC remains unclear.

In this study, GSE68799, GSE53819, and GSE103611 datasets were downloaded from the Gene Expression Omnibus Database (GEO) to analyze the expression and prognosis of m^6^A-related genes on NPC. Immunohistochemical analysis (IHC) was used to detect the expression of m^6^A-related genes in NPC tissues to determine the prognostic biomarkers of NPC.

## Materials and Methods

### Public Data Source

The raw data and corresponding clinical information were downloaded from the Gene Expression Omnibus Database (GEO) (https://www.ncbi.nlm.nih.gov/geo/). The expression array data (GSE53819) containing 18 NPC tissues and 18 control tissues, the throughput sequencing data (GSE68799) containing 42 NPC tissues and 4 control tissues, and the GSE102349 dataset containing 113 NPC tissues were used for subsequent analysis.

### Differential Expression Analysis of m^6^A-Related Genes

EdgeR package was used for screening the differentially expressed genes (DEGs) between NPC tissue and control tissue with the adjusted *p* < 0.05 and |log_2_FC| > 1 from GSE53819 and GSE68799.

### Consensus Clustering Analysis

To determine whether the expression levels of m^6^A-related genes were associated with prognosis. Four m^6^A genes (METTL3, YTHDF3, IGF2BP1, and IGF2BP2) were selected for a Consensus Clustering analysis using the Consensus Cluster Plus package, and *k* = 4 seemed to be the most appropriated selection in the GSE102349 datasets. The K–M method and log-rank test was employed to calculate the progression-free survival (PFS) difference between different clusters.

### Prognostic Signatures Generation and Prediction

To determine independent prognostic factors in NPC, the relationship between PFS of NPC patients in the GSE102349 dataset and four m^6^A genes (METTL3, YTHDF3, IGF2BP1, and IGF2BP2) was analyzed using univariate Cox regression. The hazard ratio (HR) of gene larger than 1 is considered a poor prognosis for high expression, while those <1 is considered a poor prognosis for low expression. According to the minimum standard (*p* < 0.05), determine three genes (GF2BP1, IGF2BP2, and METTL3) to establish a risk model, and use the LASSO regression coefficient to calculate the risk score. Univariate and multivariate PFS analyses were applied to assess the prognostic value of clinical stage, subtype, clustering, and risk score. Receiver operating characteristic (ROC) curve was constructed to evaluate the prediction accuracy of the prognostic model. The nomogram is further used to integrate risk scores, staging, EB subtype, and differentiation to predict the probability of survival at 1 and 3 years.

### Estimation of Immune Cell-Type Fractions

In order to further reveal the relationship between risk score and immune infiltration, we first obtained DEGs with |logFC (fold change)| ≥ 1 and adj. *p* < 0.05, and revealed the immune correlation of DEGs by GO analysis and KEGG analysis. The CIBERSORT (https://cibersort.stanford.edu), an approach to quantify the relative abundance of immune cell types based on specific gene expression profiles, was used to assess the distribution (normalized to 1) of 22 immune cell types in NPC patients from GSE102349. The *z*-score was used to normalize the proportion of immune cells and then univariate Cox analysis was used to analyze the relationship between different immune cells and PFS.

### Patients and Tissue Samples

Fifty-five formalin-fixed, paraffin-embedded NPC tissue specimens and 20 normal nasopharyngeal mucosa (NNM) were collected from Xiangya Hospital of Central South University from December 2011 to January 2013. The patients' clinical stage was classified and reclassified according to the AJCC criteria (28, 29). The patients were newly diagnosed patients without radiotherapy and chemotherapy with an informed consent and were used for immunohistochemical staining. All patients received conventional radiotherapy and chemotherapy and were followed up after treatment. The follow-up period at the time of analysis was 10.33–91.67 months (median, 44.56 ± 17.56). Overall survival (OS) is the date from the first treatment to cancer-related death or, if the patient is alive, the latest examination. Disease-free survival (DFS) is calculated as the time from the completion of the initial radiotherapy to the date of pathological diagnosis or clinical evidence of local failure and/or distant metastasis. This study was approved by the ethics committee of Xiangya hospital, Central South University. The clinical and pathological characteristics of the NPC cases used in this study are shown in Table 1.

**Table 1:** Correlation between METTL3 expression and clinicopathological characteristics in nasopharyngeal carcinomas (number = 55, χ^2^-test).

**Variables**	**Number of cases**	**METTL3**		***p*-value**
		**Low**	**High**	
Gender				
Male	30	19	11	0.509
Female	25	15	10	
Age				
≤ 45	31	21	10	0.227
>45	24	13	11	
Histologic type (WHO)				
Differentiation	28	14	14	0.059
Undifferentiation	27	20	7	
Primary tumor (T) stage				
T1–2	20	9	11	0.051
T3–4	35	25	10	
Lymph node (N) metastasis				
N0	26	10	16	0.001^*^
N1–3	29	24	5	
Distant metastasis (M)				
M0	32	13	19	0.000^*^
M1	23	21	2	
Clinical TNM stage				
I–II	24	8	16	0.000^*^
III–IV	31	26	5	

* *Statistically significant*.

### Immunohistochemistry (IHC)

IHC of formalin-fixed and paraffin-embedded tissue sections according to the universal SP kit (mouse/rabbit streptavidin–biotin detection system, SP-9000, ZSBIO). Briefly, tissue sections were dewaxed and then antigen-repaired, and endogenous peroxidase blockers were added to block endogenous peroxidase activity. After blocking the antigen, the tissue sections were incubated with Anti-METTL3 antibody (ab195352, abcam) at 4°C overnight and then incubated with biotin-labeled goat anti-mouse/rabbit IgG polymer for 15 min at room temperature. Subsequently, horseradish enzyme-labeled streptavidin working solution was added to promote antigen–antibody binding. Finally, staining was performed using DAB Chromogenic Kit (ZLI-9018, ZSBIO) and hematoxylin staining solution (BA-4097, Baso) to detect immunoreactive cells. Two independent pathologists who were unaware of the clinical parameters scored the intensity of staining and the proportion of positive cells. After negotiation, the scores were as follows: Dyeing intensity: 0 for non-staining, 1 (weak) for light yellow, 2 (medium) for brown, and 3 (strong) for tan. The coloration ratio (positive cell ratio) is classified as 0 without coloring, <30% coloring as 1, 30–60% coloring as 2, and >60% coloring as 3. By adding the two scores of staining intensity and coloring ratio (range, 0–6), a score of ≤ 3 is considered as METTL3 low expression, and >3 is considered as METTL3 high expression.

### Cell Lines and Culture

High metastatic 5–8F (6, 30) and well-differentiated HK1 (31) NPC cell lines were established and kindly gifted by Dr. HM Wang of the Cancer Center, Sun Yat-sen University, China. Both cell lines were cultured in RPMI-1640 medium containing 10% FBS at 37°C in 5% CO_2_.

### Western Blot and RT-PCR

The total proteins and total RNA were extracted from NPC cells, and then Western blots and RT-PCR were used to detect the protein and mRNA levels of METTL3 in NPC cells as previous described (5, 6, 32).

### Statistics

All data were analyzed statistically using SPSS software package ver20.0 (SPSS, Inc., Chicago, IL, USA) and GraphPad Prism 7.0 (GraphPad Software, La Jolla, CA, USA). *p* < 0.05 was considered statistically significant.

## Results

### m^6^A-Related Genes Are Dysregulated and Associated With the Patient Overall Survival in NPC

**Figure 1:**
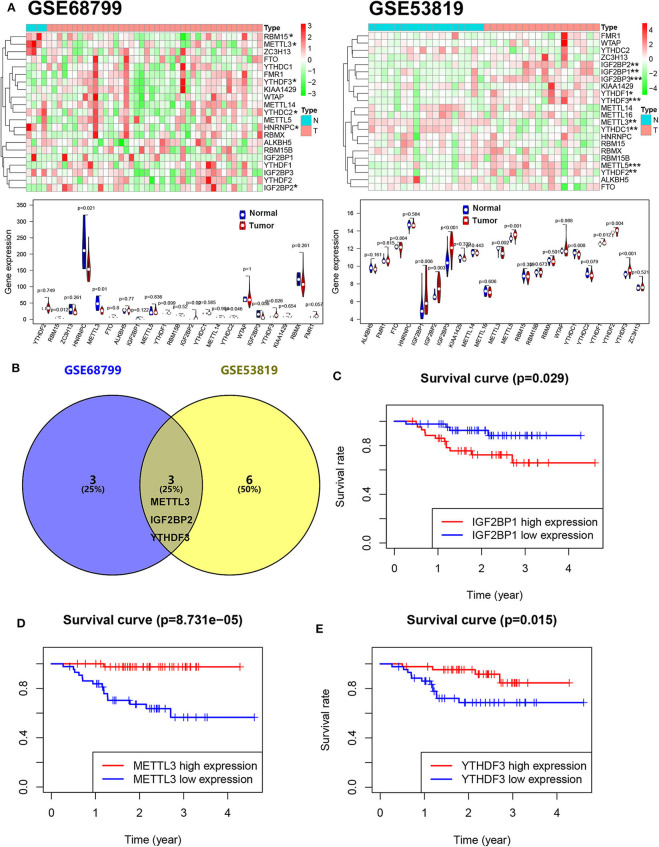
The m^6^A gene expression is associated with the PFS in NPC. **(A)** The expression levels of m^6^A-related genes in GSE53819 and GSE68799. **(B)** The overlapped differentially expressed m^6^A genes between GSE53819 and GSE68799. Kaplan–Meier curves of PFS for high- and low-expression IGF2BP1 **(C)**, METTL3 **(D)**, and YTHDF3 **(E)** in GSE102349.

To reveal the dysregulation of m^6^A-related in NPC, we downloaded an expression array data (GSE53819, containing 18 NPC tissue and 18 control tissue) and a throughput sequencing data (GSE68799, containing 42 NPC tissue and 4 control tissue) from GEO. The expression levels of m^6^A-related genes including 10 “writers” (METTL3, METTL5, METTL14, METTL16, KIAA1429, VIRMA, WTAP, RBM15, RBM15B, and ZC3H13), 12 “readers” (YTHDC1, YTHDC2, YTHDF1, YTHDF2, YTHDF3, HNRNPC, FMR1, eIF3, IGF2BP1, IGF2BP2, IGF2BP3, and RBMX), and 2 “erasers” (FTO and ALKBH5) were analyzed. As shown in Figure 1A, four genes (METTL3, RBM15, HNRNPC, and YTHDC2) were downregulated and two genes (IGF2BP2 and YTHDF3) were upregulated in NPC tissue compared to normal tissue in GSE68799. Moreover, two genes (METTL3 and YTHDC1) were downregulated and seven genes (YTHDF1, YTHDF2, METTL5, YTHDF3, IGF2BP2, IGF2BP3, and IGF2BP1) were upregulated in NPC tissue compared to normal tissue in GSE53819. Two upregulated genes (YTHDF3 and IGF2BP2) and a downregulated gene (METTL3) are overlapped in GSE68799 and GSE53819 (Figure 1B). We next analyzed the relationship between the 22 m^6^A genes' expression and PFS of patients with NPC in GSE102349. The results showed that high expression of IGF2BP1 and low expression of METTL3 and YTHDF3 showed poor PFS in NPC patients (Figures 1C–E and Figure S1).

### Consensus Clustering of m^6^A Genes in Four Clusters With Different Clinical Outcomes of NPC

**Figure 2:**
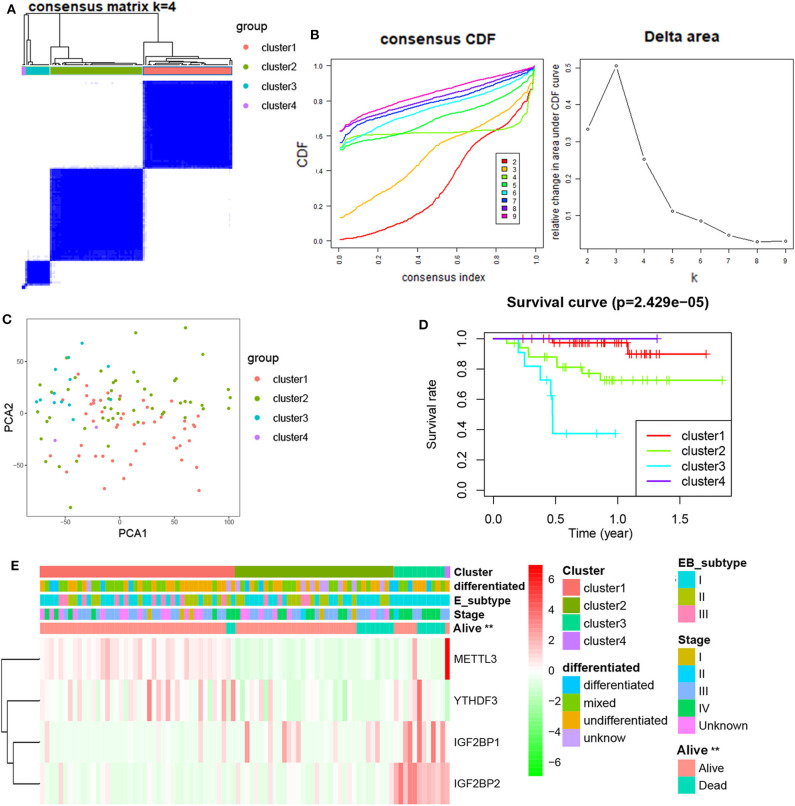
Consensus Clustering of m^6^A-related genes in NPC. **(A)** Consensus clustering matrix for *k* = 4; **(B)** consensus clustering cumulative distribution function for *k* = 2–9; **(C)** PCA analysis of four clusters in NPC; **(D)** Kaplan–Meier curves of PFS for four clusters in NPC; **(E)** the heatmap of and clinicopathologic features of four clusters of m^6^A-related genes.

Next, differentially expressed or prognostic m^6^A genes (METTL3, YTHDF3, IGF2BP1, and IGF2BP2) were selected for a Consensus Clustering analysis using the Consensus Cluster Plus package, and *k* = 4 seemed to be the most appropriated selection in the GSE102349 datasets (Figures 2A,B). The PCA results showed that four clusters could be better distinguished (Figure 2C). A significant difference of PFS was observed among four clusters (Figure 2D). Moreover, METTL3 and YTHDF3 were high expression in the cluster 1 subgroup, IGF2BP1 was highly expressed in the cluster 2 and 3 subgroup, and IGF2BP2 was highly expressed in the cluster 3 subgroup. The cluster subgroup is significantly correlated with living state, but not associated with the clinical stage, differentiation, and expression-based subtype (Figure 2E).

### Prognostic Analysis of Risk Model and m^6^A Genes

**Figure 3:**
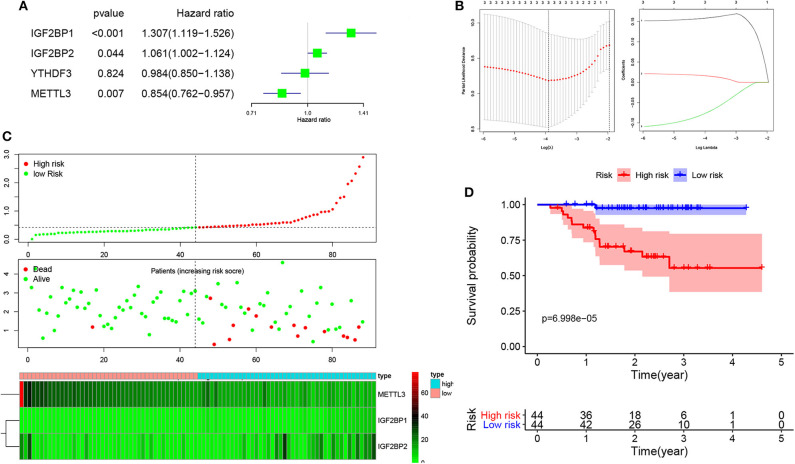
Risk model from three m^6^A-related genes. **(A)** Univariate Cox analysis revealed the relationship between four m^6^A genes and PFS of NPC patients. **(B)** The risk model calculated by LASSO. **(C)** The distributions of risk scores, alive/dead status, and expression of three m^6^A-related genes. **(D)** Kaplan–Meier PFS curves for patients in high/low risk.

To develop an m^6^A-related gene signature for prognosis prediction of NPC, the univariate Cox regression analysis was used to reveal the relationship between four m^6^A genes (METTL3, YTHDF3, IGF2BP1, and IGF2BP2) and the PFS of NPC patients in the GSE102349 datasets. As shown in Figure 3A, the NPC patients with high expression of IGF2BP1 (HR = 1.307, 95% CI = 1.119–1.526) and IGF2BP2 (HR = 1.061, 95% CI = 1.002–1.124) and low expression of METTL3 (HR = 0.854, 95% CI = 0.726–0.957) have a poor prognosis. Next, based on the minimum criteria, three genes (GF2BP1 + IGF2BP2 + METTL3) were used to build the risk model, and the coefficients were used to calculate the risk score using LASSO regression (Figure 3B). The risk score = IGF2BP1 × 0.161557 + IGF2BP2 × 0.01313 + METTL3 × (−0.0624). The risk score distribution, survival status, and expression profile of the four prognostic MDEGs are shown in Figure 3C. The patients with high risk have a poorer PFS in NPC (Figure 3D).

### Risk Score Is an Independent Prognostic Factor in NPC

**Figure 4:**
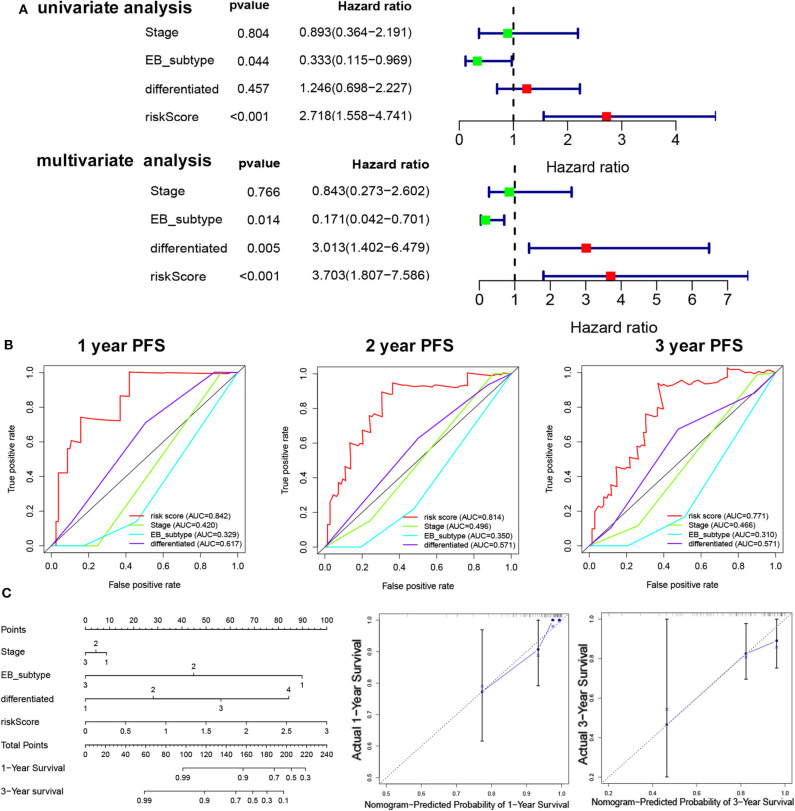
The independent prognostic factor in NPC. **(A)** Univariate/multivariate COX regression analyses of clinicopathologic factors associated with NPC. **(B)** The ROC curve of clinicopathologic factors. **(C)** Establishment of the PFS nomogram for NPC patients.

To determine the independent prognostic factor in NPC, univariate, and multivariate PFS analysis were used to evaluate the prognostic value of clinical stage, subtype, cluster, and risk score. The univariate analysis showed that the subtype (*p* = 0.044, HR = 0.333, 95% CI = 0.155–0.969) and risk score (*p* < 0.001, HR = 2.718, 95% CI = 1.558–4.741) were significantly correlated with the PFS. The multivariate Cox analysis revealed the risk score (*p* < 0.001, HR = 2.274, 95% CI = 1.280–4.039), EB subtype (*p* = 0.014, HR = 0.171, 95% CI = 0.0042–0.701), and differentiation (*p* = 0.005, HR = 3.013, 95% CI = 1.402–6.479) as the independent prognostic factor (Figure 4A). The relationship between risk score and clinicopathologic factors is also observed in Figure S2. The ROC curve analysis showed that the risk score model had a better efficiency in predicting 1-, 2-, and 3-year OS, with the AUC value being 0.84, 0.81, and 0.77, respectively (Figure 4B). Next, nomogram was used for integrating risk score, stage, EB subtype, and differentiation to predict the probability of survival at 1 and 3 years (Figure 4C). The calibration curves revealed that predictive curves were close to the ideal curve (Figure 4C).

### DEGs From High/Low-Risk Group in NPC

**Figure 5:**
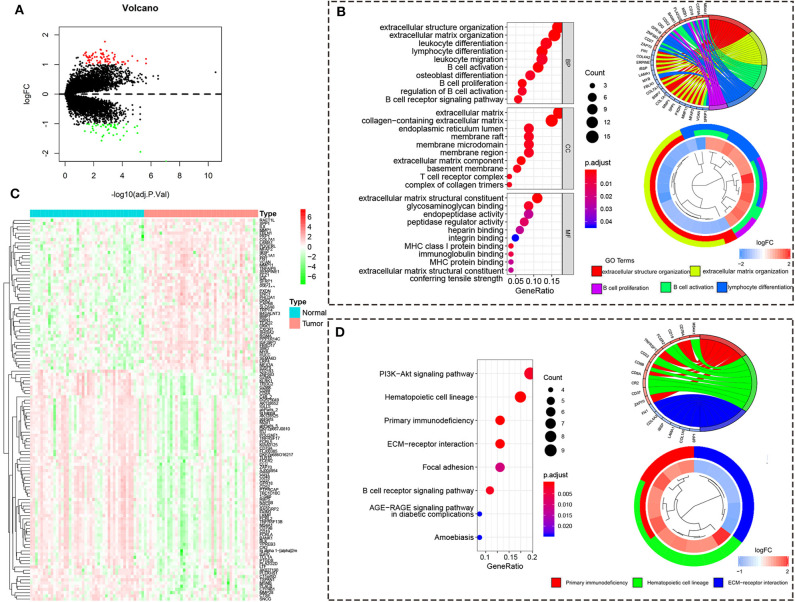
The differentially expressed gene from the high/low-risk group. **(A)** The volcano map of DEGs from the high/low-risk group. Red means upregulated genes, and blue means downregulated genes. **(B)** The heatmap of DEGs from the high/low-risk group. **(C)** The GO analysis of DEGs. **(D)** The KEGG analysis of DEGs.

To reveal the function regulated by high/low risk, we obtained the DEGs with the |logFC (fold change)| ≥ 1 and adj. *p* < 0.05. There are 72 upregulated DEGs and 47 downregulated DEGs in the high-risk group compared to the low-risk group (Figure 5A). The heatmap of 119 DEGs was visualized in Figure 5B. The GO analysis showed that the DEGs are enriched in immune response including B cell activation, lymphocyte differentiation, B cell proliferation, leukocyte differentiation, leukocyte migration, and so on (Figure 5C). The KEGG analysis showed that the DEGs are enriched in primary immunodeficiency, PI3K-Akt signaling pathway, B cell receptor signaling pathway, and so on (Figure 5D).

### Risk Score Is Associated With Immune Landscape in NPC Patients

**Figure 6:**
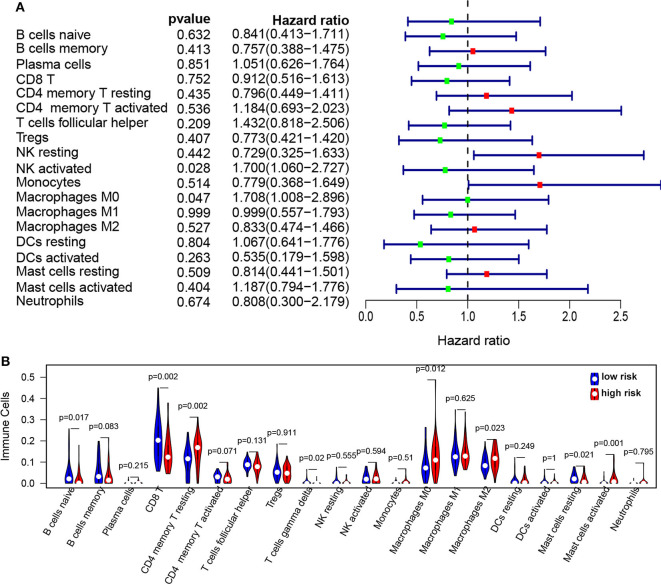
The infiltration of immune cells in the high/low-risk groups in NPC. **(A)** Forest plots revealed the relationship between immune infiltration and PFS in NPC. **(B)** The immune cell infiltration in high-risk groups compared to low-risk groups.

To further reveal the relationship between risk score and immune infiltration, the immune cells in NPC patients were estimated using the CIBERSORT method. The proportion of immune cells in NPC patients were observed in Figure S3. The proportion of immune cells were normalized using *z*-score, and then univariable Cox analysis was used for the relationship between immune cells and PFS. We found that NK-activated cell (*p* = 0.027, HR = 1.7, 95% CI = 1.06–2.73) and Macrophages M0 (*p* = 0.046, HR = 1.7, 95% CI = 1.01–2.89) were significantly correlated with the PFS (Figure 6A). The infiltration of immune cells (B naïve cells, B memory cells, CD8 T, CD4 memory T activated, T gamma delta, Mast cells resting, and activated Mast cells) was significantly increased and the infiltration of immune cells (CD4 memory T resting, Macrophages M0, and Macrophages M2) was significantly decreased in the high risk score group compared to low risk score group (Figure 6B). Moreover, correction analysis showed that METTL3 expression was correlated with infiltration of various immune cells, including B naïve cells, B memory cells, CD8 T cells, Macrophages M0, activated Mast cell, and resting CD4 memory T cell (*p* < 0.05; Figure S4).

### METTL3 Expression in NPC Tissue

**Figure 7:**
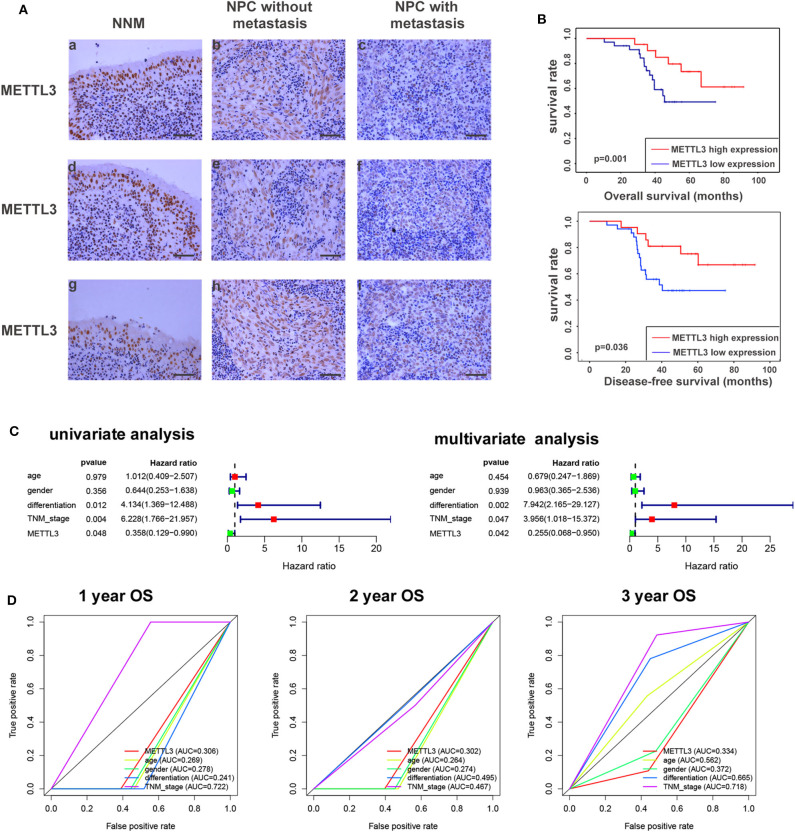
METTL3 expression in nasopharyngeal carcinoma tissue. **(A)** Immunohistochemistry showed the expression level of METTL3 in NNM **(A,D,G)**, non-metastatic NPCs **(B,E,H)**, and metastatic NPCs **(C,F,I)**. The figure shows three replicate results. **(B)** Kaplan–Meier OS curves (up) and DFS curves (below) for patients in METTL3 high/low expression. **(C)** Univariate/multivariate COX regression analyses of clinicopathologic factors associated with NPC. **(D)** The ROC curve of clinicopathologic factors.

Given the key role of METTL3 in the m^6^A modification process and potential role of METTL3 in NPC (Figure S5). We detected the expression of METTL3 in NPC tissue using IHC. Compared with the NNM tissue, METTL3 expression was downregulated in NPC tissues (*p* < 0.01), and METTL3 expression was lower in metastatic NPC tissues than in non-metastatic NPC tissues (*p* < 0.01), indicating that METTL3 expression is downregulated during the occurrence and metastasis of NPC (Figure 7A, Figure S6, and Table 2). The analysis of the correlation between METTL3 expression level and clinical pathological characteristics of NPC showed that METTL3 expression level was negatively correlated with TNM stage (*p* = 0.000) and metastasis (*p* = 0.000; Table 1). K–M analysis showed that METTL3 expression level was negatively correlated with the OS (*p* = 0.001) and DFS of NPC patients (*p* < 0.05; Figure 7B). Subsequently, univariate and multivariate OS analysis evaluated the prognostic value of age, gender, differentiation, TNM stage, and METTL3. The univariate analysis showed that the differentiation (*p* = 0.012, HR = 4.134, 95% CI = 1.369–12.488), TNM stage (*p* = 0.004, HR = 6.228, 95% CI = 1.766–21.957), and METTL3 (*p* = 0.048, HR = 0.358, 95% CI = 0.129–0.990) were significantly correlated with the OS. The multivariate Cox analysis revealed the differentiation (*p* = 0.002, HR = 7.942, 95% CI = 2.165–29.127), TNM stage (*p* = 0.047, HR = 3.956, 95% CI = 1.018–15.372), and METTL3 (*p* = 0.042, HR = 0.255, 95% CI = 0.068–0.950) as the independent prognostic factor (Figure 7C). The ROC curve analysis showed that METTL3 showed a better efficiency in predicting 1-, 2-, and 3-year OS, with the AUC value being 0.306, 0.302, and 0.344, respectively (Figure 7D). The above results indicate that low METTL3 expression is associated with poor prognosis of NPC.

**Table 2:** The expression of METTL3 in the normal nasopharyngeal mucosa and nasopharygeal carcinoma tissues.

	**NNM**	**NPC**
METTL3		
Low (0–3)	4	34
High (4–6)	16	21

p = 0.001 by the χ^2^-test, NNM vs. NPC.

*NNM, normal nasopharyngeal mucosal tissues; NPC, nasopharyngeal carcinoma tissues*.

## Discussion

NPC is one of the head and neck malignancies with geographical distribution. Previous studies have shown that the progression of NPC is regulated by epitranscriptomics (33, 34). m^6^A is the most common and plentiful modification in post-transcriptional modification of RNA, but so far, little is known about the role of m^6^A in NPC. Based on the genomic analysis dataset (GSE68799, GSE53819, and GSE102349), YTHDF3, IGF2BP2, and METTL3 were dysregulated in NPC. Subsequently, prognostic analysis showed that IGF2BP1 correlate inversely and METTL3 and YTHDF3 correlate positively with poor PFS in NPC patients. Consensus Clustering analysis revealed four subgroups with significant differences for PFS. Subsequently, the risk signature using three genes (GF2BP1 + IGF2BP2 + METTL3) was shown to be an independent prognostic marker of NPC.

Accumulating studies showed that the tumor immune microenvironment plays a vital role in the progression and prognosis of various cancers (35). NPC is characterized as a highly immunogenic tumor characterized by high rates of tumor-infiltrating lymphocytes (TILs) (36, 37). Programmed death ligand 1 (PD-L1) expression was also reported to be correlated with poor outcomes of NPC (38, 39). Targeting the immune checkpoint pathway was an immunotherapeutic strategy for NPC (40). In this study, GO and KEGG analysis revealed that the risk model was significantly associated with immune-related signal pathway. The infiltration of immune cells (B naïve cells, B memory cells, CD8 T, CD4 memory T activated, T gamma delta, Mast cells resting, and activated Mast cells) was significantly increased and the infiltration of immune cells (CD4 memory T resting, Macrophages M0, and Macrophages M2) was significantly decreased in the high risk score group compared to the low risk score group. These results indicated that m^6^A genes are involved in NPC progression partly via regulating immune microenvironment.

Our risk signature proves that METTL3 is low-expressed in NPC, negatively correlated with OS, and related to the infiltration of various immune cells. METTL3 is one of the most important m^6^A methyltransferases (41), and its abnormal expression can change the fate of m^6^A transcripts, thereby affecting the proliferation, differentiation, and metastasis of various tumor cells. METTL3 was reported to be overexpressed in glioblastoma (42, 43), and with METTL3 as a tumor suppressor, overexpression of METTL3 can inhibit the growth and self-renewal of glioblastoma stem cells (GCS) (21). At present, the relationship between METTL3 and NPC is unclear. Although Zhang et al. (44) concluded that METTL3 is highly expressed in NPC, they detected the METTL3 expression in head and neck squamous cell carcinoma (HNSC) from the Cancer Genome Atlas (TCGA) data set incorrectly, and used quantitative RT-PCR technology to prove that METTL3 mRNA was highly expressed in NPC cell lines. There are no data to prove the protein levels of METTL3 in NPC tissue and cells. Our study found that in the genomic analysis datasets (GSE68799, GSE53819, and GSE102349), METTL3 was low-expressed in NPC and correlated with the prognosis of NPC patients. Immunohistochemical analysis revealed that METTL3 protein was low-expressed in NPC tissues. Meanwhile, we also detected the expression level of METTL3 in metastatic and non-metastatic NPC tissues. The mRNA and protein levels of METTL3 were also detected in high metastatic 5–8F cells and well-differentiated HK1 cells (Figure S7). The protein levels of METTL3 were higher in HK1 cells than 5–8F cells. No difference of METTL3 mRNA was observed between HK1 cells than 5–8F cells. The results showed that METTL3 expression was negatively correlated with metastasis. Survival analysis showed significant differences in survival between patients with low and high METTL3 expression, showing a negative correlation. These results indicate that METTL3 is a risk factor for NPC metastasis and m^6^A mRNA methylation machinery may be as a promising therapeutic target for NPC (21).

Moreover, we found that IGF2BP1 and YTHDF3 (two readers of the m^6^A modification) have inverse correlation with patients' survival rate in CHOL. A recent study showed that IGF2BP1 strengthens m^6^A recognition on RNAs, such as c-Myc mRNA, to increase the mRNA stability and expression of c-Myc, thereby promoting tumorigenesis (45). So, we speculated that IGF2BP1 may affect the progression of CHOL via increasing the mRNA stability of some oncogene genes. Zheng et al. (46) showed that YTHDF3 as an m^1^A reader decreased invasion and migration of trophoblast by inhibiting IGF1R expression. IGF1R was also reported as an oncogene in cancers (47). So, we speculated that YTHDF3 may regulate the expression of some oncogenes in CHOL. However, these speculations need to be confirmed by further experiments.

In summary, our study has demonstrated dysregulation, prognosis, and potential function of m^6^A gene in NPC, and verified the low expression and clinical significance of METTL3 in NPC using immunohistochemical techniques. Our results indicate m^6^A genes as a promising prognostic biomarker for NPC and provide novels insight for NPC therapeutic strategies.

## Data Availability Statement

Publicly available datasets were analyzed in this study. This data can be found here: The raw data and corresponding clinical information were downloaded from Gene Expression Omnibus Database (GEO; https://www.ncbi.nlm.nih.gov/geo/). The expression array data (GSE53819) containing 18 NPC tissue and 18 control tissue and the throughput sequencing data (GSE68799) containing 42 NPC tissue and 4 control tissue and the GSE102349 dataset containing 113 NPC tissue were used for subsequent analysis.

## Ethics Statement

The studies involving human participants were reviewed and approved by the ethics committee of Xiangya School of Medicine, Central South University. The patients/participants provided their written informed consent to participate in this study.

## Author Contributions

SL and YZ conceived and designed the experiments. SL and ZY performed the experiments. ZX, SL, and ZY analyzed the data. YZ prepared the figures and/or tables.

## Conflict of Interest

The authors declare that the research was conducted in the absence of any commercial or financial relationships that could be construed as a potential conflict of interest.
