# Supplementary material for: Gene Signatures and Prognostic Values of m6A Genes in Nasopharyngeal Carcinoma
Source: Front Oncol. 2020 Jun 11;10:875. doi: 10.3389/fonc.2020.00875 (PMC7300221; doi:10.3389/fonc.2020.00875)
Supplement: Supplementary file 1 [file Data_Sheet_1.docx]

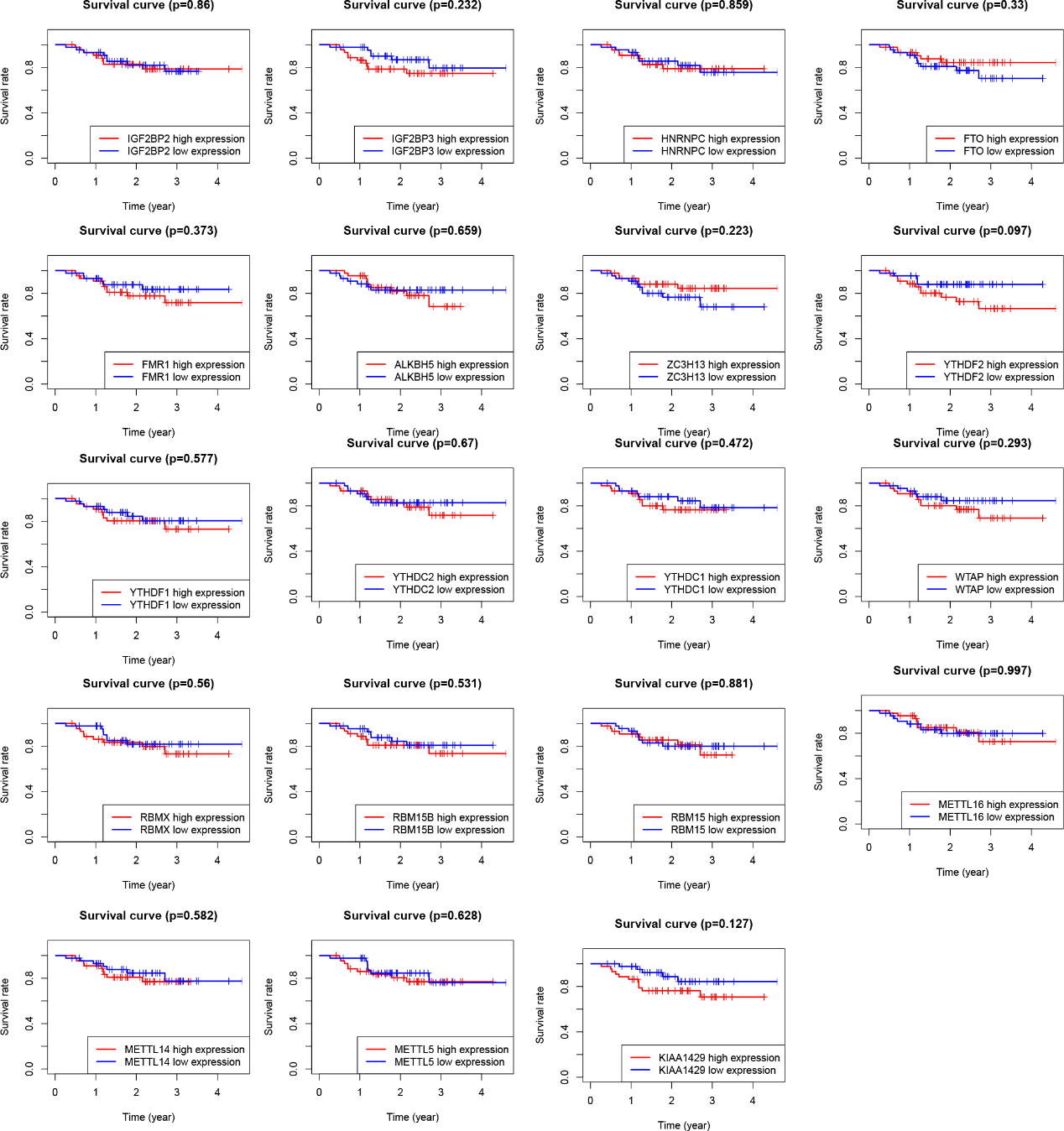


Figure S1. Kaplan–Meier curves of PFS for high and low expression of m6A related genes.


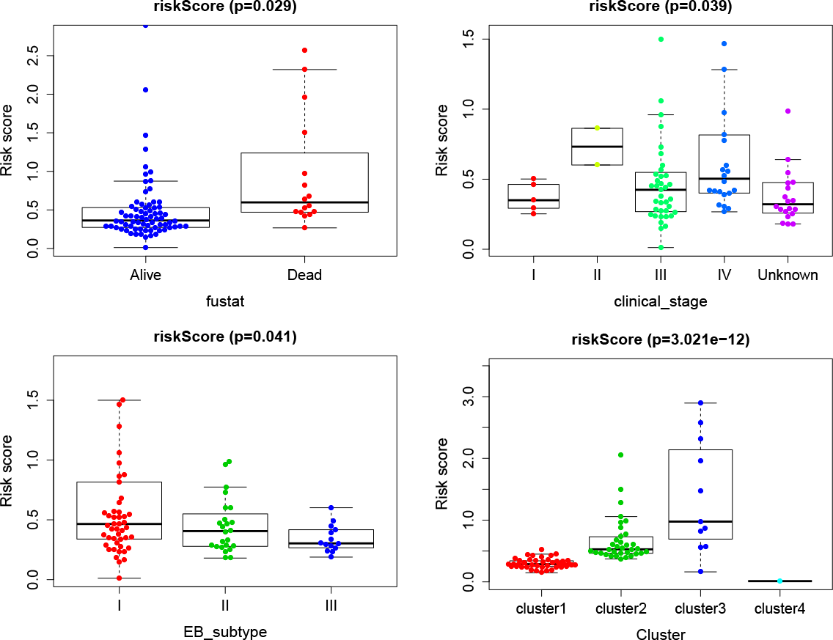


Figure S2. The relationship between risk score and **clinicopathologic factors.**


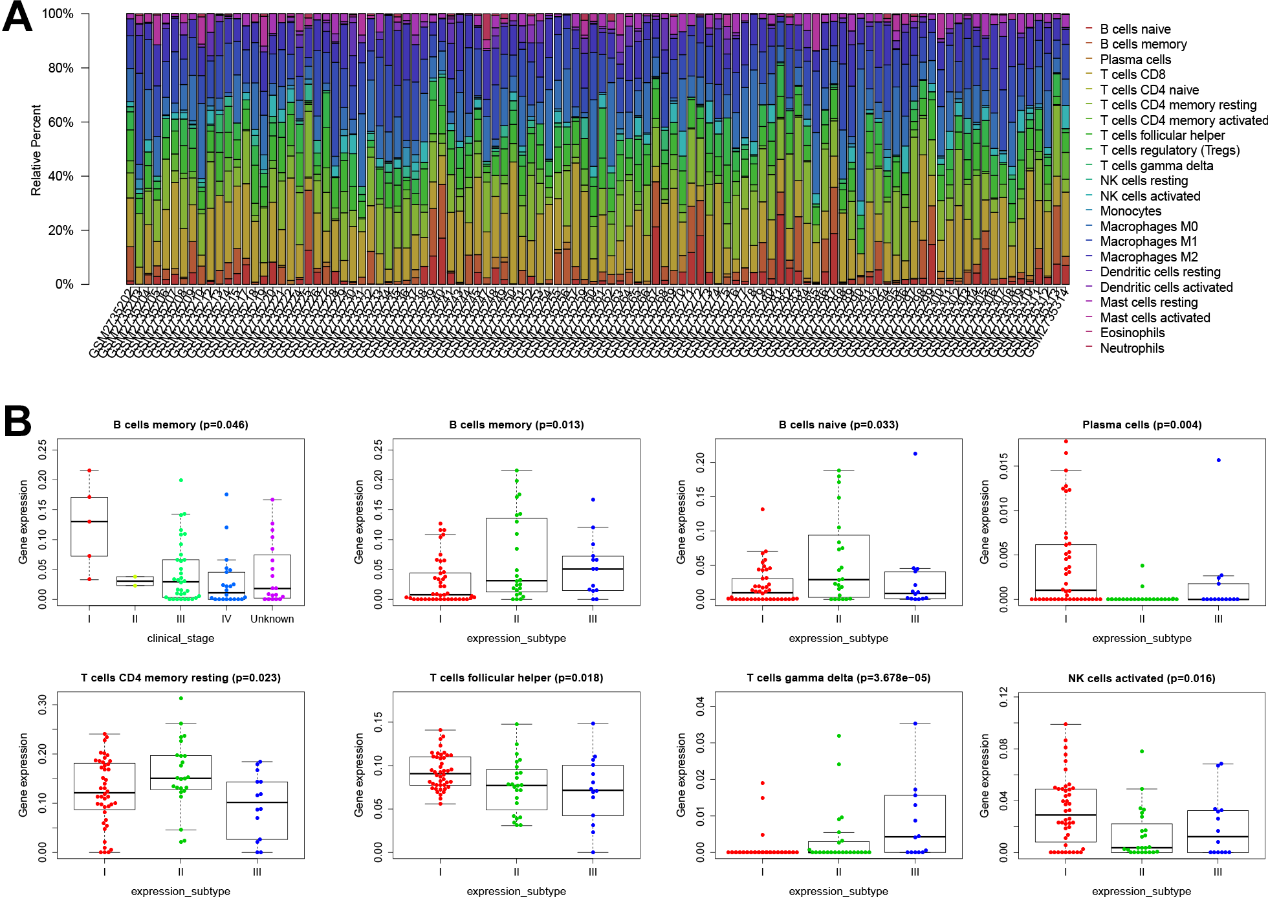


Figure S3. The immune infiltration in NPC patients. **(A)** Relative proportion of immune cells in NPC. **(B)** The relationship between immune infiltration and **clinicopathologic factors.**


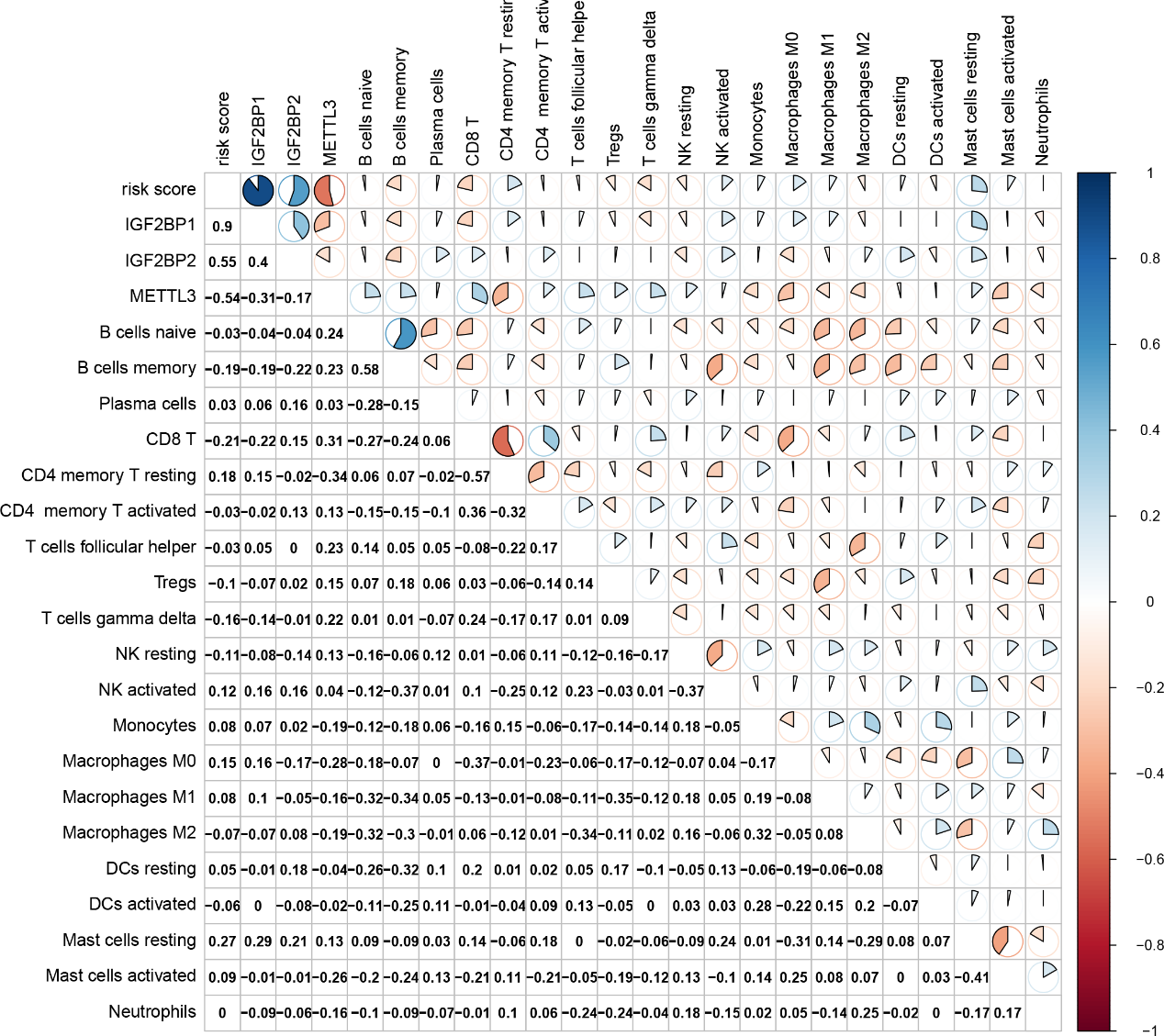
 Figure S4. METTL3 expression was correlated with infiltration of various immune cells.


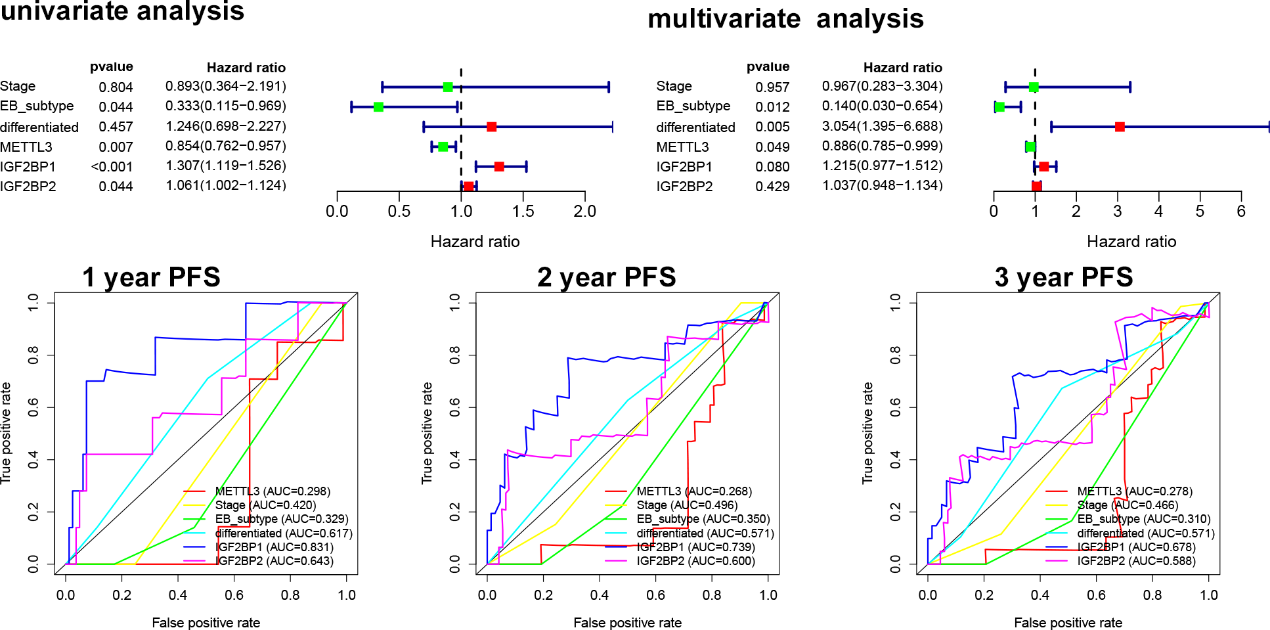


Figure S5. Univariate/multivariate COX regression analyses of m6A genes and clinicopathologic factors associated with NPC, the ROC curve of m6A genes and clinicopathologic factors in NPC in GSE102349 datasets.


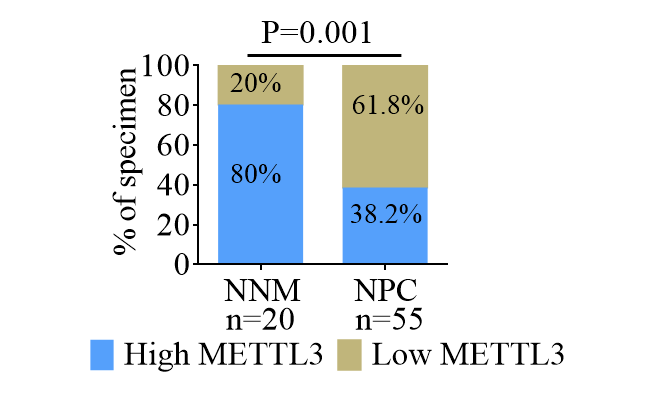


Figure S6. The expression of METTL3 in the normal nasopharyngeal mucosa and nasopharygeal carcinoma tissues


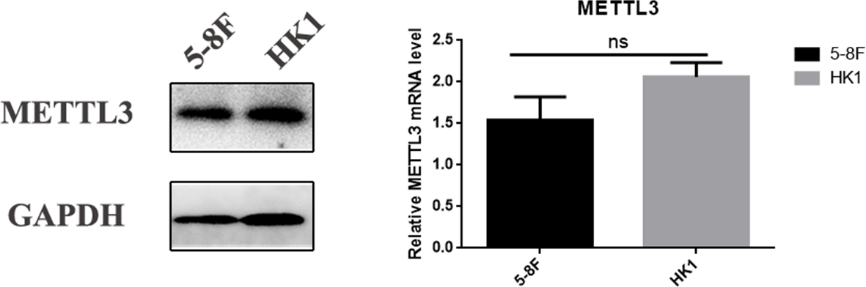


Figure S7. The protein and mRNA expression of METTL3 in two NPC cell lines.
